# Detection of an ExPEC-like *Escherichia coli* ST536 Isolated from Depurated Retail Mussels: Genomic Insights into Food Safety and One Health Surveillance

**DOI:** 10.3390/biotech15030061

**Published:** 2026-07-31

**Authors:** Ricardo J. Figueiredo, Guilherme Moreira, Ana Machado, Eliane Silva, Adriano A. Bordalo, João R. Mesquita

**Affiliations:** 1 School of Medicine and Biomedical Sciences (ICBAS), University of Porto, 4050-313 Porto, Portugal; up202206087@up.pt (R.J.F.); gmoreiravet@gmail.com (G.M.); elianepimenta05@gmail.com (E.S.); bordalo@icbas.up.pt (A.A.B.); jrmesquita@icbas.up.pt (J.R.M.); 2 Interdisciplinary Centre of Marine and Environmental Research (CIIMAR), University of Porto, 4450-208 Matosinhos, Portugal; 3 Research Center in Biodiversity and Genetic Resources (CIBIO/InBIO), University of Porto, 4485-661 Vairão, Portugal; 4 BIOPOLIS Program in Genomics, Biodiversity and Land Planning, CIBIO, Campus de Vairão, 4485-661 Vairão, Portugal; 5 Department of Chemistry and Biochemistry, Faculty of Sciences, LAQV, REQUIMTE, University of Porto, 4050-313 Porto, Portugal

**Keywords:** *Escherichia coli*, ExPEC-like, *Mytilus galloprovincialis*, antimicrobial resistance, One Health, whole-genome sequencing, food safety, depuration

## Abstract

Filter-feeding bivalves can accumulate fecal bacteria and antimicrobial resistance determinants, yet routine regulatory monitoring relies mainly on quantitative *Escherichia coli* criteria. This study provides a whole-genome characterization of an ExPEC-like *E. coli* ST536 (O21) isolate recovered from commercially depurated retail mussels originating from a Class B harvesting area in Portugal. The isolate was phenotypically identified by MicroScan WalkAway Plus and subjected to antimicrobial susceptibility testing and Oxford Nanopore long-read sequencing, followed by genome assembly, annotation, antimicrobial resistance and virulence screening, plasmid and prophage detection, and phylogenomic analysis. Phenotypically, only resistance to tobramycin was detected using the MicroScan WalkAway system. Genomic analysis revealed a complex mobile element-rich architecture, including multiple resistance-associated determinants, 116 virulence-associated loci, prophage regions, and a mobilizable IncY plasmid carrying CTX-like class A β-lactamase-associated hits detected by ABRicate. Despite its ST536 assignment and ExPEC-associated traits, phylogenomic analysis placed the isolate within a phylogroup A/commensal-like background rather than among classical B2 ExPEC lineages, suggesting acquisition of pathoadaptive modules by an atypical environmental lineage. These findings highlight the potential utility of WGS as a complementary tool and suggest that depurated bivalves may serve as sentinels, warranting further investigation.

## 1. Introduction

Global aquaculture reached a record 130.9 million tonnes in 2024, with the European Union (EU) contributing approximately 1.0 million tonnes, corresponding to an estimated value of 4.6 billion euros [1]. Within the EU, mussel cultivation represents a major aquaculture sector, accounting for 32.8% of the total live weight of farmed aquatic organisms [1,2].

In 2019, Portugal reported the highest per capita seafood consumption in the European Union (59.9 kg), with 89% of the population consuming these products at least once per week [3]. Although seafood is widely perceived by Portuguese consumers as an important component of a healthy diet, awareness of associated biological risks and environmental impacts remains limited [3]. This is particularly important in coastal countries like Portugal, where the nation food footprint is strongly influenced by fish and seafood consumption, in contrast with other Mediterranean countries where meat is the main driver [3]. Furthermore, with 77% of residents purchasing seafood through supermarkets, the potential for wide-scale exposure to foodborne pathogens and resistant genetic determinants through modern commercial supply chains can be particularly relevant [3]. Consequently, the intersection of high consumption rates and a centralized distribution system makes the monitoring of bivalves a top priority for regional food safety [3].

While the scale of the mussel aquaculture industry provides important ecosystem services, including nutrient removal, mitigation of eutrophication, and improvement of coastal water, the biological nature of mussels as filter feeders introduces a complex public health challenge [4]. As filter-feeding organisms, mussels concentrate particulate matter and microorganisms from the surrounding water column, including bacteria of faecal origin and other potentially hazardous biological agents [5,6,7]. Consequently, mussels may act both as bioindicators of environmental contamination and as vehicles for microbial exposure through the food chain.

Among bacteria associated with faecal contamination, *Escherichia coli* remains the primary regulatory indicator to assess the microbiological quality of live bivalve molluscs intended for consumption [8].

*E. coli* colonizes the gastrointestinal tract of humans and warm-blooded animals acting as commensal, often associated with symbiotic associations with their host [9,10]. However, specific lineages have acquired virulence factors that enable them to cause intestinal or extraintestinal severe disease [10]. Among these, extraintestinal pathogenic *E. coli* (ExPEC) are of particular concern due to their zoonotic and clinical relevance [10,11]. Differently from the diarrheagenic pathotypes, ExPEC are specialized strains capable of colonizing the host outside the gut [10]. ExPEC lineages are the leading cause of urinary tract infections (UTIs), bloodstream infections (sepsis), neonatal meningitis, pneumonia and soft tissue infection, representing an important and growing burden on global healthcare systems [10,12].

Phylogenetic analysis classifies *E. coli* into eight groups: A, B1, B2, C, D, E, F, G [13,14]. ExPEC strains are frequently associated with specific phylogenetic backgrounds and virulence-associated gene profiles. Historically, ExPEC lineages have been mainly linked to phylogroups B2 and D, whereas commensal strains are more commonly associated with phylogroup A and B1 [12].

However, the population structure of *E. coli* is diverse and has been revised over time, with additional phylogroups now recognized [13,14]. Therefore, phylogenetic classification, sequence typing, and virulence profiling are important for understanding the potential origin, host association, and public-health relevance of environmental- and food-associated *E. coli* and also for monitoring the spread of multidrug resistance [12].

Within ExPEC-associated lineages, Sequence Type 536 (ST536) has been described as a classical and uropathogenic globally disseminated ExPEC clone [15], historically recognized as the prototype for the study of pathogenicity islands (PAIs) [15]. The classic ST536 genomic backbone is characterized by robust virulence machinery, making it a high-risk lineage in clinical settings, highlighting the importance of its identification and monitoring [15].

Antimicrobial Resistance (AMR) is currently recognized as a major global public health threats, and its transmission extends beyond clinical settings to include animals, food systems, wastewater, and aquatic environments [16]. Within this framework, antibiotic-resistant *E. coli*, particularly third generation cephalosporin resistant and carbapenem resistant strains, is included in the WHO Bacterial Priority Pathogens List, reinforcing the public health importance of monitoring resistant *E. coli* beyond clinical settings [17]. Coastal environments may act as important interfaces for the circulation of resistant bacteria and antimicrobial resistance genes. In this context, filter feeding bivalves are especially relevant because they can accumulate bacteria and genetic determinants from contaminated waters and may contribute to their transfer along the food chain [8,12]. In the European Union, microbiological criteria for live bivalve molluscs are mainly based on quantitative microbiological criteria (≤230 MPN/*E.coli*/100 g), as established by the European Commission Regulation (EC) No 2073/2005 [18]. However, these criteria assess hygienic quality and do not require genomic characterization of *E. coli* isolates for antibiotic resistance genes, virulence factors, plasmids, prophages, or other mobile genetic elements. Traditional microbiological methods, such as culture base detection and antimicrobial susceptibility testing (AST), remain the routine standard for identifying AMR bacteria and are essential for food safety monitoring; however, these approaches are limited to phenotypic profiling [19]. In contrast, modern molecular approaches, such as whole genome sequencing (WGS), enables high-resolution genetic information regarding resistance determinants that conventional AST cannot capture, allowing for the precise tracking of high-risk clonal lineages crossing the environmental–clinical boundary [19]. Although *E. coli* is routinely used as an indicator of fecal contamination, studies conducted in different geographical and sanitary contexts have demonstrated that bivalves may harbor strains with clinically relevant virulence or antimicrobial resistance traits. In three French coastal shellfish-harvesting areas classified as category B for mussels and oysters and category C for cockles, STEC and EPEC strains were recovered from shellfish and their associated watersheds. Subsequent characterization of 28 STEC and 75 EPEC isolates identified subsets carrying virulence profiles suggestive of potential pathogenicity to humans [20]. Similarly, in natural estuarine shellfish banks in southeastern Brazil, *E. coli* was detected in 32% of 150 mussel and oyster samples, and six of the confirmed isolates were classified as EPEC. In Atlantic Canada, 27% of the *E. coli* isolates recovered from oysters and mussels carried virulence traits consistent with pathogenic extraintestinal lineages, while antimicrobial-resistant isolates were detected at sites located close to untreated human effluent sources. More recently, ESBL-producing and multidrug-resistant *E. coli* have also been reported in mussels obtained from natural and retail sources [21,22]. Collectively, these findings indicate that the occurrence and characteristics of clinically relevant *E. coli* in bivalves may vary according to geographical setting, fecal pollution sources, harvesting conditions, and post-harvest handling. They also demonstrate that quantitative enumeration of *E. coli* alone does not distinguish commensal isolates from strains carrying pathogenicity- or resistance-associated traits. Under the European law, specifically the Commission Implementing Regulation (EU) 2019/627, bivalve mollusc harvesting areas are strictly classified [23]. While Class A bivalves ≤230 MPN of *E. coli* per 100 g of shellfish flesh and intravalvular liquid [SFIL] are safe for direct human consumption, Class B (>230 to ≤4600 MPN *E. coli*/100 g of SFIL, in 90% or more of samples) requires purification in a depuration facility or short-term relaying [20]. Class C (>4600 to ≤46000 MPN *E. coli*/100 g of SFIL) bivalves, being more contaminated, cannot be placed on the market after standard depuration and strictly require long-term relaying (at least two months in clean marine waters) or a rigorous commercial heat treatment to eliminate pathogens (Articles 53, 54 and 55) [23].

Despite increasing concern regarding antimicrobial resistance in marine ecosystems and food-associated environments, the occurrence and genomic characterization of clinically relevant *E. coli* lineages from commercially available mussels remain poorly explored, particularly in Portugal. Consequently, mussels that are fully compliant with current legal safety limits current microbiological safety standards [17] may still act as silent vehicles for high-risk, drug-resistant lineages reaching the consumer. To address this critical gap, this study aims to provide a comprehensive genomic characterization of an ExPEC-like *Escherichia coli* ST536 lineage recovered from a Class B harvesting area and obtained at retail after commercial depuration. By employing Whole Genome Sequencing (WGS), we seek to demonstrate that bivalves fully compliant with current European microbiological safety standards can still act as silent reservoirs for high-risk clones carrying mobilizable resistance determinants and robust virulence machinery of public health relevance. Ultimately, this research highlights the limitations of relying solely on traditional culture-based indicator counts and underscores the urgent need to integrate high-resolution genomic surveillance into routine seafood safety protocols within a One Health framework.

## 2. Materials and Methods

### 2.1. Sample Collection

The isolate analysed in this study belonged to a previously established collection of bacteria recovered from retail mussels purchased in Portugal since January 2016. A single 1 kg retail package of fresh, chilled Mediterranean mussels (*Mytilus galloprovincialis*), corresponding to one commercial batch (lot 5292), was obtained from a commercial retail outlet in Northern Portugal. According to the product label, the mussels originated from Spanish aquaculture and were packed on 24 February 2018. The product had undergone commercial depuration before retail placement and originated from a Class B harvesting area. Following purchase, the package was transported to the laboratory in an insulated container maintained at approximately 4 °C. A pooled portion comprising approximately 25 g of mussel flesh and intravalvular liquid was used for microbiological analysis. The product was therefore considered compliant with the microbiological criteria required for sale and consumption.

### 2.2. Sample Processing

The mussel tissues were homogenized under aseptic conditions. A representative portion of the sample. Approximately 25 g of mussel homogenized tissue and intra-valvular liquid was weighed into a sterile stomacher bag. The sample was then diluted with 225 mL of Buffered Peptone Water (BPW), to achieve a 1:10 (*w*/*v*) initial dilution [24]. The mixture was processed using a peristaltic homogenizer (Stomacher) at high speed for 120 s to ensure complete tissue disintegration and release of the microbial load [25].

### 2.3. Bacterial Identification and Antimicrobial Susceptibility Testing

The resulting primary homogenate flasks were incubated for 18 h at 35 °C. After enrichment with BPW, an aliquot of the surface growth was inoculated onto MacConkey agar (Merck, Darmstadt, Germany) and incubated at 37 °C for 24 h [26]. The isolate was subsequently preserved at −80° in Luria–Bertani broth with 25% glycerol until further analysis.

Following incubation, a colony showing morphology compatible with *E. coli* was selected and streaked onto fresh MacConkey agar to obtain a pure culture. Culture purity was assessed based on uniform colony morphology and confirmed by Gram staining. Bacterial identification and minimum inhibitory concentration (MIC) determination were performed using the MicroScan WalkAway Plus system with Neg-Urine-Combo 98 panels (Beckman Coulter, Tokyo, Japan). All procedures followed the manufacturer instructions and aligned with the European Committee on Antimicrobial Susceptibility Testing (EUCAST) and Clinical and Laboratory Standards Institute (CLSI) guidelines for antimicrobial susceptibility testing (AST) interpretation [27].

The Neg-Urine-Combo 98 panel comprised 25 antimicrobial agents distributed across several antimicrobial classes. The β-lactam agents included ampicillin (4–8 µg/mL), amoxicillin–clavulanic acid (8/2 and 32/2 µg/mL), piperacillin–tazobactam (8/4–16/4 µg/mL), and aztreonam (1–4 and 16 µg/mL). The cephalosporins evaluated were cefuroxime (8 µg/mL), cefoxitin (8 µg/mL), cefotaxime (1–16 µg/mL), ceftazidime (1–16 µg/mL), and cefepime (1 and 4–8 µg/mL), together with cefotaxime–clavulanic acid (0.25/4–0.5/4 and 4/4 µg/mL) and ceftazidime–clavulanic acid (0.25/4–0.5/4 and 2/4 µg/mL). Carbapenem testing included ertapenem (0.12–0.5 µg/mL), imipenem (2–4 µg/mL), and meropenem (0.12 and 2–8 µg/mL). The quinolone group comprised nalidixic acid (16 µg/mL), ciprofloxacin (0.06 and 0.25–1 µg/mL), levofloxacin (0.5–1 µg/mL), and norfloxacin (0.5–1 µg/mL), whereas the aminoglycosides tested were amikacin (8–16 µg/mL), gentamicin (2–4 µg/mL), and tobramycin (2–4 µg/mL). The remaining agents were colistin (2–4 µg/mL), fosfomycin (8 and 32 µg/mL), nitrofurantoin (64 µg/mL), and trimethoprim–sulfamethoxazole (2/38–4/76 µg/mL). The panel was used according to the manufacturer’s instructions and as previously described [26], and MIC interpretations were assigned according to EUCAST criteria [27].

### 2.4. Isolate DNA Extraction

For genomic DNA extraction, the isolate was inoculated into brain heart infusion (BHI) (Liofilchem, Roseto degli Abruzzi, Italy), followed by bead mill homogenization using a TissueLyser (Qiagen, Hilden, Germany) system [28]. The isolate was incubated in 5 mL of BHI (37 °C, 24 h) before being centrifuged at 4000 rpm for 10 min. After discarding 4 mL of the supernatant, the pellet and residual medium were transferred to a 2 mL tube with acid-washed (1M HCl) 0.1 mm Precellys beads. The sample was processed in a TissueLyser at 15 Hz for 3 min for cell disruption. Genomic DNA was extracted using the Mag-Bind^®^ Universal Pathogen Core Kit and quantified via Qubit™ fluorometry using the 1× dsDNA HS Assay Kit (ThermoFisher Scientific, Waltham, MA, USA).

### 2.5. Bacterial Whole-Genome Sequencing

Genomic DNA with a concentration exceeding 50 ng/µL was submitted to Plasmidsaurus (London, UK) for long-read bacterial genome sequencing. Sequencing was carried out on an Oxford Nanopore Technologies PromethION 24 platform equipped with an R10.4.1 flow cell. Libraries were generated using the Native Barcoding Kit 96 V14 (SQK-NBD114.96; Oxford Nanopore Technologies, Oxford, UK) in accordance with the manufacturer’s protocol [29]. Basecalling of the raw FASTQ data was performed in super-accurate mode using ont-dorado-for-promethion v7.4.12, with a minimum Q-score cutoff of 10. MinKNOW v6.0.4 was used for the initial removal of adapters and barcode sequences. Read-quality metrics were subsequently examined with NanoPlot v1.43.0 [30]. Residual adapter and barcode sequences were removed using Porechop v0.2.4 [31], after which NanoFilt v2.8.0 [30] was applied to retain reads with a minimum mean quality score of 10 [32].

### 2.6. Whole-Genome Analysis

The isolate long-read data underwent initial quality filtering with Filtlong v0.2.1, removing the bottom 5% of reads [32]. An assembly was generated using Miniasm v0.3 after down sampling the reads to 250 Mb [33]. Then the data was refined to 110× genome coverage, prioritizing the removal of low-quality reads using Filtlong with a high mean quality weight (—mean_q_weight 10). The filtered reads were assembled using Flye v2.9.1 [34], with parameters optimized for high quality ONT reads. Assembly polishing was performed with Medaka v1.8.0 [35] using the high-quality reads from the previous step. The quality of the final assembly was evaluated using QUAST v5.2.0 to determine key genomic metrics [36].

Furthermore, to confirm the taxonomic identity of the isolate, Average Nucleotide Identity (ANI) analysis was conducted using pyANI v.0.2.12 against reference genomes [37].

For further genomic characterization, in silico serotyping (O and H antigens) and Sequence Type (ST) were determined using MLST v2.0 and SerotypeFinder, while the phylogenetic group was identified using the ClermonTyping (v2.1) method according to the Clermont scheme [38,39,40].

After the genomes assemble, they were annotated using Bakta V.1.12.0 full database [41].

Contamination and assemble quality were addressed with CheckM V.1.2.5 [42].

Additionally, plasmid-related sequences were analyzed using PlasmidFinder 2.1 [43], and the MOB scan server [44]. Antimicrobial resistance and virulence factor-related genes were screened using ABRicate v1.0.0 with the ResFinder [45], National Center for Biotechnology Information (NCBI) [46], and Comprehensive Antibiotic Resistance Database (CARD) [47] databases for AMR genes, and the Virulence Factor Database (VFDB) for VF-associated genes [48]. The draft genome sequence of *E. coli* was deposited in GenBank under the BioProject accession PRJNA1464419 and with the accession number JBYPZB010000001.1.

Annotated genomes were analysed with Panaroo v1.5.2 under strict filtering settings to generate a high-confidence pangenome [49]. The filtered core-genome alignment produced by Panaroo was then used for maximum-likelihood phylogenomic inference with IQ-TREE2 v2.4.0 [50]. Model selection based on the Bayesian Information Criterion identified GTR+F+I+G4 as the best-fitting nucleotide substitution model. Branch support was evaluated using 1000 ultrafast bootstrap replicates. The final phylogenetic tree was visualized and annotated in iTOL v7 [51], with isolate source information incorporated to facilitate interpretation of the observed clustering patterns. Prophage identification and annotation were performed using two complementary tools. The assembled genomes were first analysed using the PHASTER [52]. Additionally, VirSorter2 V.2.2.3 was utilized to screen for viral sequences using default parameters [53].

## 3. Results

### 3.1. Sequencing Statistics, Assembly Metrics and Circularity

Oxford Nanopore sequencing generated 202,057 reads, corresponding to a total of 528,537,481 bases. The mean and median read lengths were 2615.8 bp and 731 bp, respectively, with a read-length N50 of 9994 bp. The mean read quality score was 17.1, and the median quality score was 19.4. All reads had quality scores above Q10, while 84.9% and 44.1% of the reads exceeded Q15 and Q20, respectively.

### 3.2. Phenotypic Identification and Antimicrobial Susceptibility Testing (AST)

The Gram-negative isolate JBYPZB010000001.1 was phenotypically identified as *E. coli* with a 99.99% probability using the MicroScan WalkAway Plus system (Beckman Coulter, Tokyo, Japan) with the Neg-Urine-Combo 98 panel.

The Antimicrobial Susceptibility Testing (AST) was also employed in the same step, with the isolate showing phenotypical resistance to one of the antimicrobials tested as shown in Table S1 in the Supplementary Material.

According to EUCAST guidelines [27], the isolate only showed resistance to Tobramycin (To).

### 3.3. Isolate Bacterial Chromosomal Genome Characteristics and Classification

The isolate was classified by ANI with >99% similarity as *E. coli*, confirming the MicroScan WalkAway Plus system identification.

The de novo assembly yielded a primary chromosomal sequence, designated as JBYPZB010000001.1, with a total length of 4,814,466 bp and a GC content of 50.91%. Genome quality assessment using CheckM v1.2.5 confirmed the integrity of the assembly, classifying this isolate with an estimated contamination level of approximately 2%.

**Figure 1:**
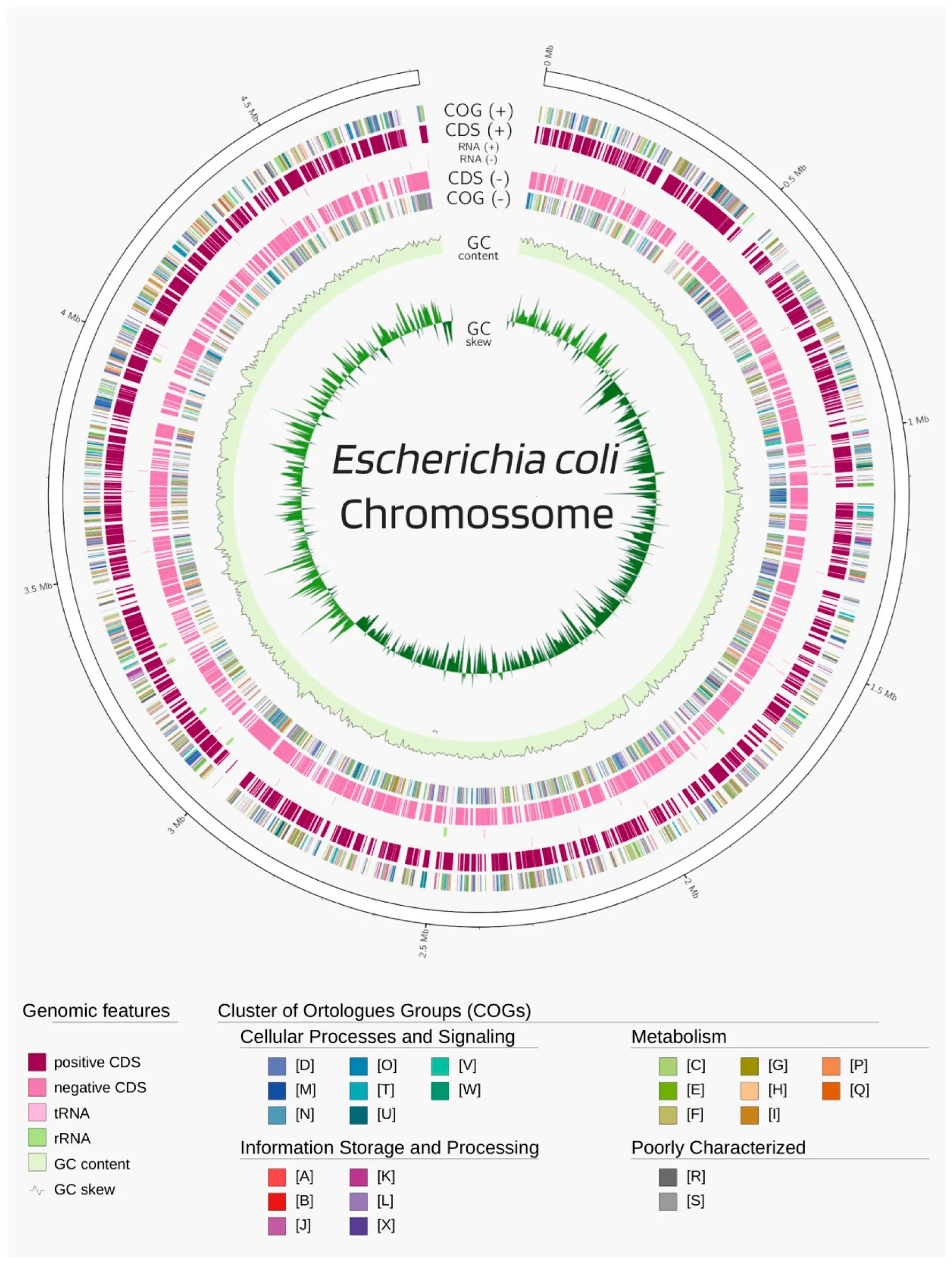
Circular genomic representation of the *E. coli* ST536 (O21:H9) chromosome. The figure displays genomic features and functional classifications based on the Cluster of Orthologous Groups (COGs).

The isolate was assigned to phylogroup A0 (*chu*A, *yja*A, *tsp*E4C2 non-present) and sequence type ST536 (serotype O21:H9) using Clermontyper v2.1 and MLST v2.0, respectively. The functional genomic annotation provided by Bakta V.1.12.0 is illustrated in Figure 1.

### 3.4. Genomic Analysis of the Isolate for Antimicrobial Resistance Genes (ARGs)

To understand the genetic basis behind the isolate phenotypic resistances, the *E. coli* genome obtained in the present study was screened against several major databases (CARD, NCBI AMRFinderPlus, ResFinder, ARG-ANNOT, and MEGARes) using ABRicate. Genome screening detected 51 AMR-associated loci, sharing between 98% and 100% identity with known sequences. However, most corresponded to intrinsic chromosomal efflux systems, regulatory genes, membrane-associated determinants, or housekeeping loci rather than acquired ARGs. No canonical acquired aminoglycoside resistance determinant was identified that could explain the observed tobramycin-resistant phenotype.

Most detected resistance-associated genes were chromosomally located and correspond to intrinsic efflux systems, permeability-associated determinants, regulatory genes, or housekeeping resistance-associated loci. These included genes associated with multidrug efflux systems, such as *acrA*, *acrB*, *tolC*, and several *mdt* family genes, as well as regulatory genes including *marA*, *marR soxS*, and *robA* [54]. These genes are listed in Table 1.

The AMR-associated loci including identity, coverage, genomic location, coordinates, intactness, and predicted phenotype are displayed in Table S2.

### 3.5. Genomic Analysis of the Isolate for Virulence Factors

Genomic analysis of the *E. coli* isolate revealed a genomic virulence, motility, and fitness determinants localized specifically within the chromosome. To elucidate the pathogenic potential encoded within this genome, it was annotated and it was identified genetic *loci* against established reference repositories, primarily using the Virulence Factor Database (VFDB). In total, 116 virulence-associated genetic *loci* are described in this isolate, which were categorized by their biological roles and structural organization (Table 2). It includes multiple surface adhesins, such as Curli fibers and Type 1 fimbriae, alongside a complete flagellar and chemotaxis apparatus, and a highly efficient enterobactin-mediated iron acquisition system.

### 3.6. Genomic Analysis of the Isolate for Prophages

In silico analysis of the *E. coli* isolate genome revealed the presence of mobile genetic elements, specifically prophage sequences, integrated within the host chromosome. Using prophage prediction algorithms, such as PHASTER, a total of 10 putative prophage regions were identified (Table 3). Among these, three regions were classified as fully intact and potentially functional, harbouring comprehensive sets of phage-related structural, replication, and lysis genes (e.g., integrases, transposases, terminases, and capsid proteins). Two regions were classified questionable, and the remaining five were classified as incomplete.

### 3.7. Genomic Analysis of the Isolate for Plasmids and Their Characteristics

**Figure 2:**
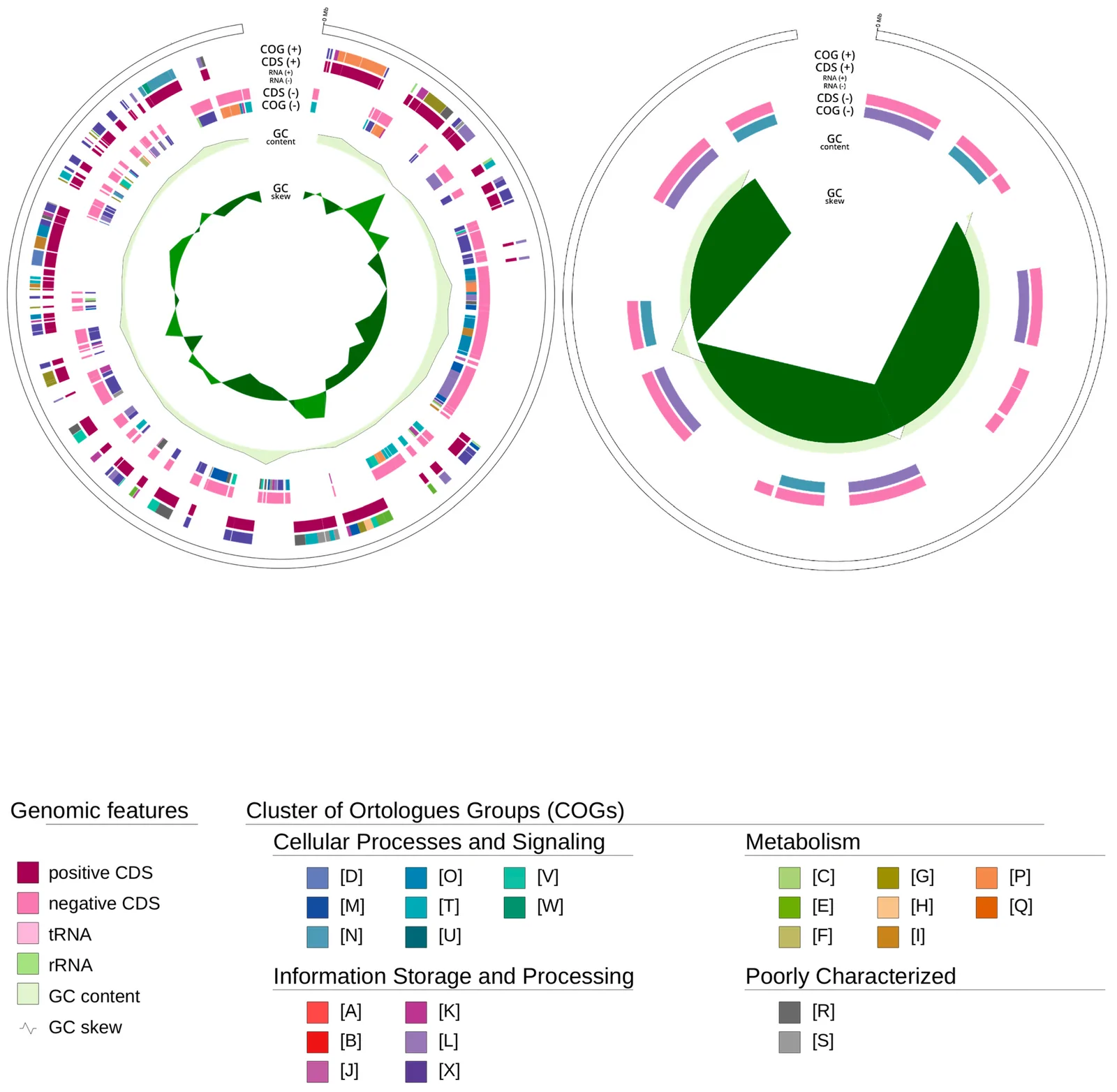
Circular representation of two genetic elements present in this genome: JBYPZB010000002.1 (**left**) and JBYPZB010000004.1 (**right**). The rings detail coding sequences, non-coding RNAs, GC content, and GC skew, with genes color-coded based on their functional classification according to the Clusters of Orthologous Groups (COG) database.

Genomic architecture and mobile genetic elements (MGEs) were characterized using MOB-suite. The main chromosome (JBYPZB010000001.1) demonstrated a high degree of plasticity, carrying numerous insertion sequences from the IS*3*, IS*5*, IS*30*, and IS*66* families. Notably, the plasmid I (JBYPZB010000002.1-209,383 bp) was identified as a mobilizable *IncY* plasmid. This element is equipped with MOBH and MOBP relaxases and a MOBF-type *oriT*, allowing it to be mobilized among *Enterobacterales*. This plasmid is heavily populated with MGEs, including IS*5075*, IS*903B*, and the Tn*3*-family transposon IS*Pa38*, underscoring its role as a dynamic vehicle for gene transfer. Additionally, minor genomic fragments were identified, including, JBYPZB010000004.1, which exhibited an atypically low GC content (32.7%), highly indicative of recent exogenous acquisition [21]. The JBYPZB010000002.1 harbours multiple copies of the *bla*_CTX-M_ gene, which encodes Extended-Spectrum Beta-Lactamases (ESBLs). However, the isolate was phenotypically susceptible to the β-lactams tested, including third-generation cephalosporins and carbapenems. Therefore, the functional relevance of these β-lactamase-associated determinants requires further confirmation. These findings are displayed in Figure 2.

The analysis of the extrachromosomal compartments revealed that JBYPZB010000002.1 harboured three prophage-associated regions, most notably a 60.3 Kb intact sequence (Score 150) exhibiting high homology to the P1-like *Enterobacteria* phage, alongside two additional regions classified as questionable and incomplete. These findings are displayed in Table 4.

### 3.8. Phylogenomic Analysis

**Figure 3:**
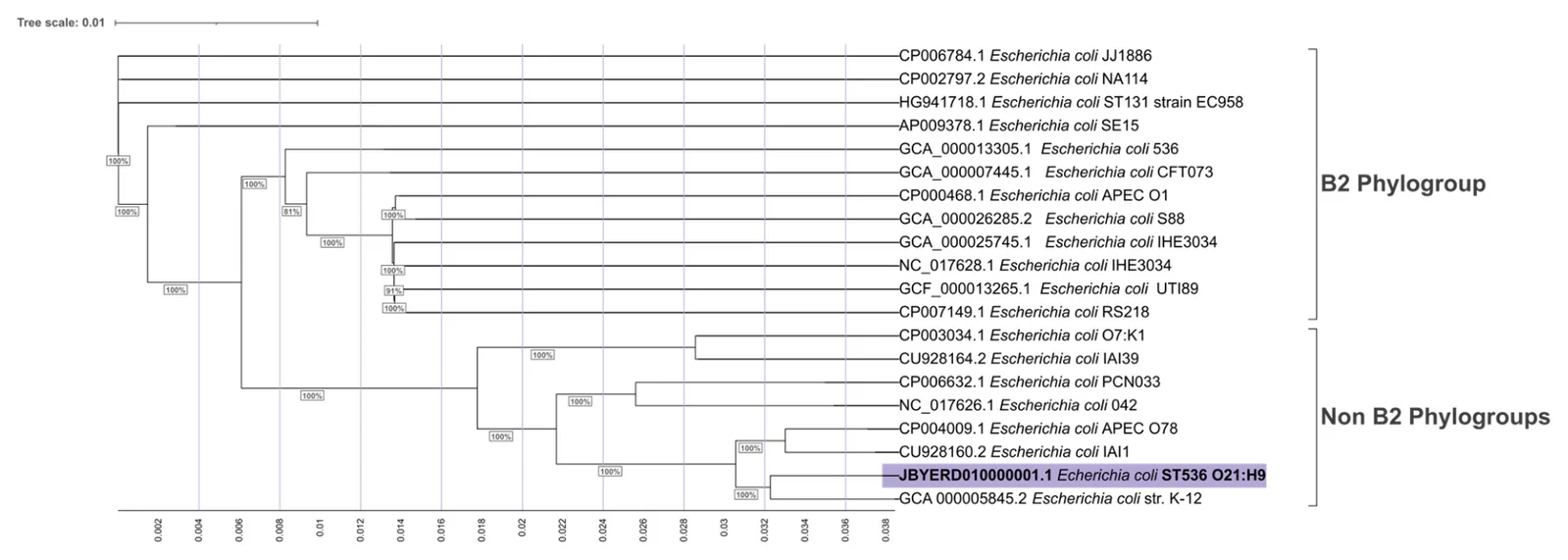
Phylogenomic placement of *E. coli* ST536 O21:H9 (JBYPZB010000001.1) isolate in this study. Phylogenetic tree was constructed with the *E. coli* ST536 O21:H9 isolate against other well-characterized *E. coli* strains using the maximum-likelihood method. Nodal support is indicated by proportional grey circles representing bootstrap values (81–100%). *E. coli* ST536 O21:H9 (JBYPZB010000001.1) isolate in this study is highlighted in bold. The reference genome information is displayed in Supplementary Table S3.

To determine the evolutionary relationship and pathotype lineage of the clinical isolate *Escherichia coli* ST536 O21:H9, a phylogenetic reconstruction was performed against a curated dataset of reference genomes, encompassing extraintestinal pathogenic (ExPEC), uropathogenic (UPEC), neonatal meningitis (NMEC), globally disseminated pandemic clones, and commensal lineages. The resulting phylogenetic tree (Figure 3) showed topological stability, with most primary branching nodes exhibiting high bootstrap support (81–100%), indicating strong statistical confidence in the lineage separation. The phylogeny distinctly segregated the analyzed genomes into two major evolutionary branches. The upper clades were predominantly occupied by highly virulent ExPEC lineages and pandemic clones. This included a distinct, well-supported cluster of the multiresistant ST131 clones, such as JJ1886, NA114, and EC958, alongside a larger clade encompassing classical ExPEC reference strains. Within this broader ExPEC cluster, classical UPEC isolates, including CFT073, 536, and UTI89, and highly invasive NMEC isolates, such as S88, RS218, and IHE3034, grouped together, reflecting a shared phylogenetic backbone typically associated with the virulent phylogroup B2.

The target isolate, *E. coli* ST536 O21:H9, did not cluster with these classic extraintestinal pathotypes. Instead, it is positioned within a distinct, more basal evolutionary branch that is primarily associated with intestinal and commensal lineages. The tree reveals that the isolate formed a highly supported terminal node directly with the non-pathogenic laboratory reference strain *E. coli* str. K-12, a classic representative of phylogroup A. Furthermore, this sub-clade was positioned adjacently to other non-ExPEC reference genomes, including the commensal strain IAI1 and the enteroaggregative *E. coli* strain 042. The phylogenetic distance and strict clustering of *E. coli* ST536 O21:H9 with the K-12 reference strain demonstrate that its core genome is genetically divergent from typical high-risk ExPEC lineages. This evolutionary proximity to commensal and intestinal phylogroups suggests that any clinical virulence or extraintestinal capabilities exhibited by this O21:H9 isolate are likely not intrinsic to its core chromosomal backbone but rather driven by the acquisition of mobile genetic elements or specific pathoadaptive mutations.

## 4. Discussion

This study reports the genomic characterization of an ExPEC-like *Escherichia coli* ST536 (O21:H9) isolated recovered from depurated retail mussels originating from a Class B harvesting area in Portugal. Although the isolate originated from a food product compliant with current microbiological regulations (EU Reg. 2073/2005) including depuration before retail and consumption, whole-genome sequencing revealed a complex genomic architecture harboring virulence-associated determinants, antimicrobial resistance genes, prophages, and mobile genetic elements. These findings reinforce the role of bivalves as environmental sentinels and potential reservoirs of clinically relevant bacterial lineages within a One Health approach [7]. The recovery of an *E. coli* with ExPEC-associated traits and mobile resistance determinants from retail mussels is particularly relevant, as it suggests that current post-harvest control measures may reduce bacterial loads without necessarily eliminating isolates carrying clinically relevant genomic features. Depuration is an important post-harvest intervention intended to reduce microbial contamination in live bivalves; however, its efficacy is influenced by the bivalve species, physiological state, initial contamination level, microbial taxon, water temperature and salinity, treatment duration, and depuration-system configuration [55]. Recent evidence indicates that depuration can substantially reduce antimicrobial-resistant bacteria and resistance genes in oysters, supporting its value as a risk-mitigation measure, although complete elimination is not consistently achieved [56]. Clearance may also differ between bacterial taxa and bivalve species. For example, experimental studies have reported variable elimination of *E. coli* and other emerging bacterial pathogens, with greater persistence of *E. coli* in mussels than in oysters under comparable conditions [24]. Therefore, depuration should not be regarded as a sterilization procedure, and compliance with conventional faecal-indicator thresholds does not necessarily imply the absence of individual bacteria carrying resistance or virulence-associated determinants. The recovery of the present isolate from commercially depurated mussels is consistent with the possibility that individual antimicrobial-resistant bacteria may persist after treatment. However, because no paired pre- and post-depuration samples were analyzed, the effectiveness of the depuration process used for this batch could not be directly assessed.

This variability should also be interpreted within the context of the bivalve microbiome. Bivalves harbour complex microbial communities whose composition is influenced by host species, tissue type, environmental conditions, harvesting area, and post-harvest processing [57]. Recent microbiome-based analyses have demonstrated that depuration can markedly restructure the bacterial community of *Mytilus galloprovincialis*, altering the relative abundance and diversity of resident taxa rather than uniformly removing the microbiota [58]. Mussel digestive glands may additionally harbor diverse antimicrobial resistance genes whose distribution is associated with microbial-community composition, mobile genetic elements, and environmental factors [58]. Nevertheless, because the present study did not include paired pre- and post-depuration samples or quantitative analysis of resistance genes, the recovery of this isolate cannot directly demonstrate depuration failure. It indicates only that an *E. coli* isolate carrying the reported genomic features was detectable in a commercially depurated retail product.

Previous studies indicate that the occurrence of clinically relevant bacteria in bivalve molluscs is strongly influenced by the harvesting environment, contamination source, shellfish species, and post-harvest conditions. In three French coastal harvesting areas classified as category B for mussels and oysters and category C for cockles, STEC and EPEC were recovered from 5.0% and 8.0% of shellfish samples, respectively, demonstrating that potentially pathogenic *E. coli* may occur in classified production areas [20]. In Atlantic Canada, antimicrobial-resistant *E. coli* isolates were detected only at sites located closest to untreated human effluent, and 27% of the recovered isolates displayed virulence profiles consistent with extraintestinal pathogenic *E. coli* of human origin [21]. By contrast, an investigation of shellfish from Galician class B harvesting areas found that commercial depuration reduced *E. coli* concentrations to ≤230 MPN *E. coli*/100 g in 90.9% of mussel lots, while virulence-associated markers for the other bacterial pathogens investigated were not detected [59]. These contrasting findings indicate that the microbiological significance of *E. coli* recovered from bivalves cannot be inferred solely from harvesting-area classification or quantitative indicator counts. In this context, the present isolate should not be interpreted as evidence of the prevalence of pathogenic *E. coli* or of depuration failure; rather, it provides a genomic case demonstrating that individual isolates recovered at retail may carry clinically relevant traits that are not characterized by routine quantitative monitoring.

Despite our isolate belonging to the ST536 lineage, it was assigned to phylogroup A rather than the commonly reported B2 phylogroup [12]. This may reflect ongoing genomic diversification, recombination events, or the acquisition of ExPEC-associated virulence repertoires by atypical environmental backgrounds [60,61]. Indeed, recent ecological genomic evidence indicates that adaptation of *E. coli* to different niches may be strongly influenced by variation in the accessory genome, including plasmids and other horizontally acquired elements, rather than by phylogroup membership alone [60]. Therefore, the coexistence of a phylogroup A core-genome background with selected ExPEC-associated loci may represent a mosaic evolutionary pattern rather than a classical ExPEC genomic profile. Accordingly, the designation “ExPEC-like” is used as a cautious genomic descriptor and does not imply a confirmed extraintestinal pathogenic phenotype. Alternatively, it may reflect limitations or inconsistencies in in silico typing that should be carefully verified.

The phenotypic resistance pattern showed resistance only to tobramycin, even though the isolate harbored an extensive resistome, including multiple *bla*_CTX-M_-associated sequences located on a mobilizable IncY plasmid. No canonical aminoglycoside resistance determinant was identified that could explain the observed tobramycin resistance. Therefore, the genetic basis of this phenotype remains unresolved, highlighting a genotype–phenotype discordance that may involve regulatory or expression-dependent mechanisms not captured by the present in silico analysis. The presence of intrinsic aminoglycoside-associated efflux systems, particularly AcrD and its BaeSR-mediated regulatory network, suggests that increased efflux activity may have contributed to the observed tobramycin resistance, although this hypothesis cannot be confirmed from gene presence alone. This discordance highlights the limitations of phenotype-only surveillance approaches and suggests the existence of cryptic or potentially inducible resistance determinants capable of dissemination under selective pressure [54,62,63].

The detection of extensive adhesion, motility, chemotaxis, and siderophore-associated systems suggests that this isolate owns substantial ecological fitness, potentially facilitating persistence within aquatic environments and host-associated niches. In particular, the coexistence of biofilm-associated curli systems and iron acquisition machinery may enhance long-term environmental survival and colonization capacity.

Of note, MEGARes detected multiple CTX-like class A β-lactamase-associated hits distributed across both the chromosome (JBYPZB010000001.1) and the larger IncY-associated plasmid (JBYPZB010000002.1). However, these matches could not be reliably resolved to specific *bla*CTX-M alleles from the MEGARes output alone. Moreover, the isolate remained phenotypically susceptible to all β-lactams tested, including third-generation cephalosporins. Therefore, the CTX-like hits were interpreted as family-level sequence similarities and not as evidence of functional ESBL production. Therefore, these CTX-like hits should be interpreted cautiously and require further confirmation using allele-resolving AMR databases, targeted sequence validation, and/or functional assays. In this context, the presence of an IncY mobilizable plasmid and associated mobile genetic elements remains relevant from a One Health surveillance perspective, but the functional contribution of these β-lactamase-associated sequences to antimicrobial resistance cannot be inferred from the current data alone.

Another relevant genomic feature was the proximity between CTX-like β-lactamase-associated hits and an intact P1-like prophage region located on the IncY-associated plasmid. This region was additionally enriched in transposase-associated elements and recombination-related proteins, suggesting the presence of a dynamic mobile genetic hotspot that may contribute to genomic plasticity. Given the recognized role of P1-like bacteriophages in horizontal gene transfer within Enterobacterales, the coexistence of prophage structures, insertion sequences, and β-lactamase-associated hits may warrant further investigation regarding their potential role in the mobilization of resistance-associated sequences [62,64,65].

The detection of holin- and endolysin-associated genes within multiple prophage regions indicates partial preservation of canonical phage lytic modules [66]. Although prophage inducibility and activity were not experimentally evaluated, the retention of these structural components may suggest residual functional potential [67]. This observation raises the possibility that some prophage elements remain capable of contributing to genome remodeling and horizontal gene transfer dynamics within environmental bacterial populations [66].

The simultaneous presence of curli-associated *loci*, type 1 fimbriae systems, and the complete enterobactin siderophore machinery suggests a genomic repertoire optimized for persistence, biofilm formation, and host-associated colonization [68,69,70]. These systems are frequently implicated in ExPEC fitness and may facilitate both environmental survival and opportunistic pathogenicity [70,71].

The coexistence of prophages, insertion sequences, transposons, and mobilizable plasmids indicates a highly dynamic genome architecture under continuous evolutionary reshaping [72]. Such genomic plasticity may facilitate rapid adaptation to environmental pressures while simultaneously increasing the probability of resistance and virulence gene exchange across ecological niches [72].

The classification of this isolate as an “ExPEC-like” strain is particularly noteworthy and requires careful contextualization. While the isolate belongs to the ST536 lineage [15], a sequence type frequently associated with classical ExPEC strains, in silico typing assigned it to phylogroup A, rather than the highly virulent phylogroup B2 typically expected for classic ExPEC pathogens [12]. To resolve this apparent discrepancy, our core-genome phylogenetic analysis provided crucial evolutionary context. The reconstructed phylogeny demonstrated that *E. coli* ST536 O21:H9 is highly divergent from classic high-risk ExPEC reference lineages (such as UPEC CFT073 or NMEC RS218). Instead, it clustered within a more basal evolutionary branch, sharing a distinct and highly supported terminal node with the non-pathogenic, phylogroup A reference strain *E. coli* K-12.

Moreover, the phylogenic placement alongside commensal and non-ExPEC lineages strongly supports the hypothesis that the core chromosomal backbone of this mussel-derived isolate is not inherently that of a primary extraintestinal pathogen. The designation “ExPEC-like” is used herein in a descriptive rather than formal pathotype sense. Screening for canonical ExPEC-associated markers identified *pap*C, involved in P-fimbrial biogenesis, and *focA*, belonging to the F1C fimbrial gene cluster. The invasion-associated genes *ibe*B and *ibeC* were also detected. In contrast, *sfa*, *afa/dra*, *hlyA*, *cnf1*, *kps*, *iutA*, *iro*, and iss were not detected. Although *hlyB*, encoding a component of the haemolysin export system, was present, the absence of *hlyA* precludes interpreting this finding as evidence of a complete α-haemolysin determinant. Therefore, the isolate does not fulfil a formal molecular definition of ExPEC. In this study, the term “ExPEC-like” refers only to the presence of a limited subset of extraintestinal virulence-associated genes in an atypical phylogenetic background, namely phylogroup A0 and genomic clustering close to *E. coli* K-12, and should not be interpreted as definitive pathotype assignment.

This genomic profile suggests that the extraintestinal virulence potential of this isolate is not intrinsic to its ancestral lineage, but rather the result of ongoing genomic diversification and the horizontal acquisition of pathoadaptive modules by an otherwise atypical, environmental, or commensal background [60,72].

Current European microbiological regulations for fresh bivalve mollusks for consumption primarily focus on quantitative *E. coli* thresholds as indicators of fecal contamination [18]. However, the present findings demonstrate that isolates recovered from legally compliant products may still harbor clinically relevant virulence and resistance-associated genomic traits. Consequently, reliance exclusively on indicator counts may underestimate the biological complexity and public health significance of bacterial populations circulating within seafood supply chains [60,72].

This study has several limitations. The analysis was based on a single *E. coli* isolate recovered from retail mussels; therefore, the findings are descriptive and cannot be used to estimate prevalence or frequency of occurrence, assess persistence, quantify consumer exposure, evaluate the effectiveness of depuration, or infer the broader epidemiological and public-health risk associated with *E. coli* contamination in retail mussels. Furthermore, the absence of systematic sampling across multiple batches, retail outlets, geographical areas, and time points limits the representativeness and generalizability of the findings. A further limitation is that MIC determination was performed exclusively using the MicroScan WalkAway system and was not confirmed by an independent method, such as agar dilution or gradient diffusion. Therefore, the tobramycin resistance result should be interpreted cautiously, particularly considering the variability in interpretive breakpoints reported for this antimicrobial. Functional validation through transcriptomic, conjugation, and pathogenicity assays would further clarify the biological relevance of the identified genomic features.

Additionally, MOB-suite, PHASTER, and VirSorter2 analyses provided computational predictions of plasmid mobility and prophage regions; however, plasmid transfer, prophage inducibility, and functional gene mobilization were not experimentally assessed. Despite these limitations, the study provides a useful genomic case highlighting the potential added value of WGS in food safety and One Health surveillance. Broader studies including larger numbers of isolates, different harvesting areas, pre- and post-depuration samples, and functional validation assays are needed to determine how frequently bivalve associated *E. coli* carry clinically relevant genomic traits and whether these traits persist through depuration and retail distribution.

## 5. Conclusions

This study successfully characterized a potentially virulent ExPEC-like *Escherichia coli* ST536 (O21:H9) isolate recovered from depurated retail mussels originating from a Class B harvesting area in Portugal. Notably, this isolate was recovered from a food product that fully adhered to current European microbiological safety regulations.

The findings highlight a critical limitation in traditional surveillance; while phenotypic testing showed resistance solely to tobramycin, whole-genome sequencing identified a resistome comprising 51 antimicrobial resistance genes.

The isolate exhibited genomic plasticity, harboring 116 virulence-associated loci, intact prophages, and a mobilizable IncY plasmid carrying multiple *bla*_CTX-like_ hit sequences.

The detection of plasmid-associated mobility elements, insertion sequences, and prophage regions suggests a mobile element rich genomic background. This complex architecture indicates a highly dynamic environment optimized for horizontal gene transfer and environmental persistence.

These results reinforce the concept that filter-feeding bivalves act as highly efficient environmental sentinels, capable of accumulating and silently transmitting clinically relevant, drug-resistant lineages to consumers and serve as reservoirs to disseminate the ARGs genes to other potential pathogenic bacteria.

Current regulations relying exclusively on quantitative *E. coli* count as an indicator of fecal contamination underestimate the biological risks present in the seafood supply chain. To effectively safeguard public health, it is essential to integrate modern genomic screening into routine food safety protocols, aligning with a comprehensive One Health approach. Overall, these findings illustrate the added value of WGS for revealing clinically relevant genomic traits not captured by routine indicator-based monitoring and support further investigation of depurated bivalves as potential One Health sentinels.

## Figures and Tables

**Table 1:** Antimicrobial resistance-associated *loci* identified in the genome of the *Escherichia coli* isolate, categorized by antimicrobial class, genomic location, and predicted resistance mechanism. Putative acquired resistance-associated determinants were distinguished from intrinsic chromosomal efflux systems, regulatory genes, membrane-associated determinants, and housekeeping loci with (CARD, NCBI AMRFinderPlus, ResFinder, ARG-ANNOT, and MEGARes databases) using ABRicate.

Antibiotic Class	ARG-Associated Loci Identified	Location	Mechanism of Action
Beta-lactams (Cephalosporins/Penicillins)	*ampC*, *ampH*, *blaEC*, *PBP2* ^b^	Chromosome	Enzymatic inactivation and target modification.
ESBL (Extended-spectrum cephalosporins) *	*CTX* (multiple copies) ^a^	Chromosome and IncY-associated plasmid	Enzymatic hydrolysis.
Multidrug (MDR) (Efflux Pumps)	*acrA*, *acrB*, *tolC*, *acrD*, *acrE*, *acrF*, *acrS*, *mdtA*, *mdtB*, *mdtC*, *mdtE*, *mdtF*, *mdtG*, *mdtH*, *mdtI*, *mdtJ*, *mdtK*, *mdtM*, *mdtN*, *mdtO*, *mdtP*, *emrA*, *emrB*, *emrD*, *mdfA*, *yojI*, *msbA*, *bcr*, *kpno* ^b^	Chromosome	Active expulsion of the antibiotic from the cell.
Macrolides (e.g., Azithromycin)	*mphB*, *mdfA* ^b^	Chromosome	Phosphorylation (inactivation) and efflux.
Polymyxins/Colistin	*ugd (PmrE)*, *pmrF*, *eptA* ^b^	Chromosome	Modification of membrane Lipopolysaccharide (LPS).
Peptides/Bacitracin	*bacA*, *yojI* ^b^	Chromosome	Carrier recycling and efflux.
Others (Fosfomycin, Fluoroquinolones)	*mdtG*, *mdtH*, *emrA/B* ^b^	Chromosome	Specific efflux via MFS/RND transporters.
Resistance Regulators	*marA*, *marR*, *soxS*, *robA*, *h-ns*, *crp*, *gadW*, *cpxA*, *cpxR*, *baeR*, *baeS*, *kdpE* ^b^	Chromosome	Control of efflux pump expression.

^a^ Putative resistance-associated determinant or family-level database hit; acquisition, expression, and functional activity were not experimentally confirmed. ᵇ Intrinsic chromosomal efflux, regulatory, membrane-associated, target-associated, or housekeeping locus; not classified as an acquired ARG. * CTX-like hits were not resolved to specific *bla*_CTX-M alleles_, and the isolate remained phenotypically susceptible to the β-lactams tested.

**Table 2:** Genomic characterization of virulence, motility, and iron homeostasis systems identified in the chromosome of the *E. coli* isolate. The table enumerates the detected genetic loci, their functional organization, and their respective biological roles in bacterial pathogenesis and survival.

System/Function	Detected Gene Loci	Location	Biological and Pathogenic Role
Curli Fibers	*csgA*, *csgB*, *csgC*, *csgD*, *csgE*, *csgF*, *csgG*	Chromosome	Initial adhesion to abiotic and biotic surfaces; major structural component of the biofilm matrix.
Type 1 Fimbriae	*fimD*, *fimF*, *fimG*, *Z2200*, *Z2201*, *Z2206*	Chromosome	Specific adhesion to mannose receptors on urothelial cells; key determinant in urinary tract colonization.
Additional Fimbriae	*stgA*, *stgB*, *stgC*, *stgD*, *ycbQ*, *ycbR*, *ycbS*, *ycbT*, *ycbU*, *ycbV*, *ycbF*	Chromosome	Accessory adhesins involved in the expansion of tissue tropism and persistence in diverse host niches.
Pili and Other Adhesins	*ppdA*, *ppdB*, *ppdC*, *ppdD*, *ygdb*, *ycfz*, *ygeG*, *ygeH*, *b2854*, *orgB*, *yggr*, *nlpI*, *hofB*, *hofC*, *hofQ*, *aec15*	Chromosome	Type IV pili and pseudopili; involved in horizontal gene transfer (conjugation), twitching motility, and intercellular adhesion.
Flagellar Biosynthesis	*flgA*, *flgB*, *flgC*, *flgD*, *flgE*, *flgF*, *flgG*, *flgH*, *flgI*, *flgJ*, *flgK*, *flgL*, *flgN*, *fliA*, *fliD*, *fliE*, *fliF*, *fliG*, *fliH*, *fliI*, *fliJ*, *fliK*, *fliL*, *fliM*, *fliN*, *fliO*, *fliP*, *fliQ*, *fliR*, *fliS*, *fliT*, *fliY*, *fliZ*, *flhA*, *flhB*, *flhC*, *flhD*, *flhE*, *flk*	Chromosome	Complete apparatus for flagellar biogenesis; confers swimming motility essential for chemotaxis and invasion.
Chemotaxis and Motor	*cheA*, *cheB*, *cheM (tar)*, *cheR*, *cheW*, *cheY*, *cheZ*, *motA*, *motB*	Chromosome	Environmental signal transduction and motor torque control; directs bacterial swimming toward nutrient gradients.
Enterobactin System	*entA*, *entB*, *entC*, *entD*, *entE*, *entF*, *fepA*, *fepB*, *fepC*, *fepD*, *fepE*, *fepG*, *fes*, *entS*	Chromosome	Production and transport of high-affinity siderophores (catechols); crucial for ferric iron sequestration in nutrient-restricted environments.
Secretion Effectors	*espL1*, *espL3*, *espL4*, *espR1*, *espX1*, *espX4*, *espX5*, *Z1307 (OmpA)*	Chromosome	Translocated effector proteins; modulation of the innate immune response and alteration of host cytoskeletal architecture.
Invasion and Stress Response	*ibeB*, *ibeC*, *cadA*, *nadA*, *nadB*, *artJ*	Chromosome	Cellular invasion proteins and acid stress resistance; support metabolic fitness and intracellular survival.

**Table 3:** Identification and characterization of putative prophage regions within the *Escherichia coli* isolate chromosome. Prophage sequences were analysed and categorized as intact (score > 90), questionable (score 70–90), or incomplete (score < 70) based on sequence length, viral protein composition, and the presence of specific phage-related structural markers. The table details the genomic location, total length, completeness status, key viral genes identified, and the primary reference phage homologs for each region.

Region	Genomic Position (bp)	Length (Kb)	Completeness (Score)	Key Phage-Related Keywords Identified	Primary Phage Homologs (Most Common Hits)
**1**	Chromosome: 367708–374390	6.6	Incomplete (30)	tail, transposase	*Enterobacteria* phage BP-4795, Stx2-II
**2**	Chromosome: 589898–634544	44.6	Intact (150)	lysin, tail, portal, transposase, capsid	*Enterobacteria* phage lambda, *Enterobacteria* phage DE3
**3**	Chromosome: 628139–666257	38.1	Questionable (80)	transposase, integrase	Stx2-converting phage 1717, *Escherichia* phage RCS47
**4**	Chromosome: 1311890–1363625	51.7	Intact (150)	tail, plate, capsid, head, lysin, integrase, portal, protease	*Salmonella* phage 118970-sal3, *Enterobacteria* phage SfV
**5**	Chromosome: 1766285–1787256	20.9	Incomplete (60)	tail, integrase	*Escherichia* phage 500465-1, *Enterobacteria* phage P4
**6**	Chromosome: 1889134–1921645	32.5	Incomplete (30)	integrase	*Escherichia* phage RCS47, *Synechococcus* phage Bellamy
**7**	Chromosome: 2766366–2771888	5.5	Incomplete (30)	transposase	*Bacillus* phage vB_BtS, *Bacillus* phage G
**8**	Chromosome: 3518453–3531471	13.0	Intact (100)	transposase, tail, head, integrase	*Shigella* phage SfIV, *Pseudomonas* phage phi3
**9**	Chromosome: 4751351–4781140	29.7	Incomplete (50)	recombinase, tail, plate	*Salmonella* phage SEN34, *Enterobacteria* phage mEpX1
**10**	Chromosome: 4778762–4807660	28.8	Questionable (70)	terminase, tail	*Pectobacterium* phage ZF40, *Escherichia* phage Lys12581Vzw

**Table 4:** Identification and classification of putative prophage regions identified on the plasmid 1 (JBYPZB010000002.1) of the *E. coli* isolate. Regions were evaluated using a heuristic scoring system where scores >90 indicate an intact prophage, 70–90 denote a questionable region, and <70 represent an incomplete or cryptic prophage remnant. Region 13 represents a fully intact P1-like prophage, containing a complete set of structural and replication machinery. Primary homologs represent the top reference phage sequences identified through BLASTp searches against viral databases.

Region	Genomic Position	Length (Kb)	Completeness (Score)	Phage-Related Keywords Identified	Primary Phage Homologs (Most Common Hits)
11	JBYPZB010000002.1: 30716–53727	23.0	Questionable (80)	transposase, tail	*Escherichia* phage RCS47, *Bacillus* phage vB_BceS
12	JBYPZB010000002.1: 78284–87832	9.5	Incomplete (50)	transposase, tail, terminase	*Salmonella* phage SJ46, *Bacillus* phage JL
13	JBYPZB010000002.1: 132497–192864	60.3	Intact (150)	recombinase, plate, tail, transposase, terminase, head	*Escherichia* phage RCS47, *Enterobacteria* phage P1

## Data Availability

The original contributions presented in this study are included in the article and Supplementary Materials. Further inquiries can be directed to the corresponding author.

## Supplementary Materials

The following supporting information can be downloaded at: https://www.mdpi.com/article/10.3390/biotech15030061/s1, Table S1. Antimicrobial Susceptibility Testing (AST) results for mussel associated isolate JBYPZB010000001.1. Table S2. Genomic characterization of predicted antimicrobial resistance (AMR) and efflux determinants. The table details the identified resistance genes, alignment identity and coverage percentages, genomic location (chromosome or plasmid), specific sequence coordinates, genetic intactness, and the associated predicted resistance phenotypes. Table S3. *Escherichia coli* strains and reference genomes used in this study. The table lists the strain designations, corresponding NCBI accession numbers, and their status as either publicly available reference genomes or novel isolates sequenced in the present study.

## Abbreviations

The following abbreviations are used in this manuscript:

AMR	Antimicrobial Resistance
ARG	Antimicrobial Resistance Gene
AST	Antimicrobial Susceptibility Testing
ANI	Average Nucleotide Identity
BPW	Buffered Peptone Water
CARD	Comprehensive Antibiotic Resistance Database
CDS	Coding Sequence
CLSI	Clinical and Laboratory Standards Institute
COG	Cluster of Orthologous Groups
DNA	Deoxyribonucleic Acid
ESBL	Extended-Spectrum β-Lactamase
EU	European Union
EUCAST	European Committee on Antimicrobial Susceptibility Testing
ExPEC	Extraintestinal Pathogenic *Escherichia coli*
GC	Guanine-Cytosine
IncY	Incompatibility Group Y
iTOL	Interactive Tree of Life
MDR	Multidrug Resistance
MGE	Mobile Genetic Element
MIC	Minimum Inhibitory Concentration
MLST	Multilocus Sequence Typing
MOB	Mobilization/relaxase-associated classification
MPN	Most Probable Number
NCBI	National Center for Biotechnology Information
NMEC	Neonatal Meningitis-Associated *Escherichia coli*
ONT	Oxford Nanopore Technology
oriT	Origin of Transfer
PAI	Pathogenicity Island
QUAST	Quality Assessment Tool for Genome Assemblies
SFIL	Shellfish Flesh and Intravalvular Liquid
ST	Sequence Type
UPEC	Uropathogenic *Escherichia coli*
UTI	Urinary Tract Infection
VF	Virulence Factor
VFDB	Virulence Factor Database
WGS	Whole-Genome Sequencing
WHO	World Health Organization
