# Supplementary material for: Detection of an ExPEC-like Escherichia coli ST536 Isolated from Depurated Retail Mussels: Genomic Insights into Food Safety and One Health Surveillance
Source: BioTech (Basel). 2026 Jul 31;15(3):61. doi: 10.3390/biotech15030061 (PMC13510357; doi:10.3390/biotech15030061)
Supplement: Supplementary file 1 [file biotech-15-00061-s001.zip › biotech-4394268-supplementary.pdf]

Supplementary Materials: Detection of an ExPEC-like *Escherichia coli* ST536 isolated from depurated retail mussels: Genomic insights into food safety and One Health surveillance

Ricardo J. Figueiredo, Guilherme Moreira, Ana Machado, Eliane Silva, Adriano A. Bordalo and João R. Mesquita

Table S1. Antimicrobial Susceptibility Testing (AST) results for mussel associated isolate JBYPZB010000001.1.

Antibiotic Class	Antimicrobial Agent (Abbreviation)	MIC (μg/mL)	Interpretation
**β-lactams**	Ampicillin (AM)	≤ 4.00	S
	Amoxicillin-clavulanic acid (Aug)	≤ 8/2	S
	Piperacillin-tazobactam (P/T)	≤ 8.0	S
**Cephalosporins**	Cefuroxime (Crm)	≤ 8.00	S
	Cefoxitin (Cfx)	≤ 8.00	S
	Cefotaxime (Cft)	≤1.00	S
	Ceftazidime (Caz)	≤1.00	S
	Cefepime (Cpe)	≤ 1.00	S
**β-lactam/Inhibitor**	Cefotaxime-clavulanic acid (Cft/CA)	≤ 0.25	S
	Ceftazidime-clavulanic acid (Caz/CA)	≤ 0.25	S
**Monobactams**	Aztreonam (AZT)	≤ 1.00	S
**Carbapenems**	Ertapenem (Etp)	≤ 0.12	S
	Imipenem (Imp)	≤ 2.00	S
	Meropenem (Mer)	≤ 0.12	S
**Quinolones**	Nalidixic acid (NA)	≤ 16.00	S
	Ciprofloxacin (Cp)	≤ 0.06	S
	Levofloxacin (Lvx)	≤ 0.50	S
	Norfloxacin (Nxn)	≤ 0.50	S
**Aminoglycosides**	Amikacin (AK)	≤ 8.00	S
	Gentamicin (Gm)	≤ 2.00	S
	Tobramycin (To)	= 4	R
**Sulphonamides**	Trimethoprim-Sulfamethoxazole (T/S)	≤ 2/38	S
**Polymyxins**	Colistin (Cl)	≤ 2.00	S
**Phosphonics**	Fosfomycin (Fos)	≤ 8.00	S
**Nitrofurans**	Nitrofurantoin (Fd)	≤ 64.00	S
**---**	---	---	---
**Summary Metrics**	Total Tested: 25	S: 96% (24)	R: 4% (1)

Table S2. Genomic characterization of predicted antimicrobial resistance (AMR) and efflux determinants. The table details the identified resistance genes, alignment identity and coverage percentages, genomic location (chromosome or plasmid), specific sequence coordinates, genetic intactness, and the associated predicted resistance phenotypes.

Gene/ determinant	Identity (%)	Coverage (%)	Genomic location	Coordinates	Intactness	Predicted phenotype
mdtG	99.59	100.00	Chromosome	75 084–76 310 (−)	Intact	Potential fosfomycin and deoxycholate reduced susceptibility through MFS efflux; overexpression dependent.
mdtH	98.59	100.00	Chromosome	84 941–86 149 (−)	Intact	Potential fluoroquinolone reduced susceptibility through multidrug efflux; expression-dependent.
hns	99.52	100.00	Chromosome	236 755–237 168 (−)	Intact	Global regulator of efflux-associated genes; presence alone does not predict resistance.
marR	97.47	100.00	Chromosome	581 500–581 934 (+)	Intact	Repressor of the *mar* regulon; resistance requires regulatory alteration or altered expression.
marA	99.74	100.00	Chromosome	581 948–582 337 (+)	Intact	Potential multidrug reduced susceptibility through AcrAB activation and OmpF repression.
mdtI	99.39	100.00	Chromosome	660 966–661 295 (−)	Intact	Potential multidrug and biocide reduced susceptibility through SMR efflux.
mdtJ	99.73	100.00	Chromosome	661 282–661 647 (−)	Intact	Potential multidrug and biocide reduced susceptibility through SMR efflux.
mdtK	98.62	100.00	Chromosome	731 424–732 797 (+)	Intact	Potential multidrug reduced susceptibility through MATE efflux.
**CTX-like**	99.05	100.00	Chromosome	846 808–847 750 (+)	Intact	Putative β-lactam hydrolysis by a CTX-like class A β-lactamase.
**CTX-like**	99.05	100.00	Chromosome	1 080 775–1 081 717 (−)	Intact	Putative β-lactam hydrolysis by a CTX-like class A β-lactamase.
***ugd* (*pmrE*)**	96.14	100.00	Chromosome	1 118 120–1 119 286 (−)	Intact	Potential polymyxin and cationic-peptide reduced susceptibility through lipid A modification.
asmA	98.06	99.95	Chromosome	1 154 545–1 156 397 (−)	Likely intact	Intrinsic efflux-associated determinant; presence alone does not predict resistance.
mdtA	99.92	100.00	Chromosome	1 168 800–1 170 047 (+)	Intact	Potential aminocoumarin and multidrug efflux as part of MdtABC–TolC.
mdtB	99.71	100.00	Chromosome	1 170 047–1 173 169 (+)	Intact	Potential aminocoumarin and multidrug efflux as part of MdtABC–TolC.
mdtC	99.77	100.00	Chromosome	1 173 170–1 176 247 (+)	Intact	Potential aminocoumarin and multidrug efflux as part of MdtABC–TolC.
baeS	99.14	100.00	Chromosome	1 177 660–1 179 063 (+)	Intact	Sensor kinase regulating MdtABC and AcrD expression.
baeR	99.45	100.00	Chromosome	1 179 060–1 179 782 (+)	Intact	Response regulator promoting MdtABC and AcrD expression.
**CTX-like**	99.05	100.00	Chromosome	1 185 742–1 186 684 (+)	Intact	Putative β-lactam hydrolysis by a CTX-like class A β-lactamase.
**CTX-like**	99.05	100.00	Chromosome	1 257 225–1 258 167 (+)	Intact	Putative β-lactam hydrolysis by a CTX-like class A β-lactamase.
bcr	99.41	100.00	Chromosome	1 307 519–1 308 709 (−)	Intact	Potential multidrug and biocide reduced susceptibility through MFS efflux.
yojI	98.30	100.00	Chromosome	1 379 676–1 381 319 (−)	Intact	Potential resistance to microcin J25 and related peptides through TolC-dependent export.

***kpnO*-like**	80.70	99.64	Chromosome	1 384 350–1 385 480 (−)	Questionable /disrupted	Possible SMR efflux determinant; low identity and alignment gaps make the prediction uncertain.
pmrF	99.38	100.00	Chromosome	1 439 503–1 440 471 (+)	Intact	Potential polymyxin reduced susceptibility through Ara4N-mediated lipid A modification.
**CTX-like**	98.94	100.00	Chromosome	1 534 996–1 535 938 (−)	Intact	Putative β-lactam hydrolysis by a CTX-like class A β-lactamase.
acrD	99.39	100.00	Chromosome	1 615 829–1 618 942 (+)	Intact	Potential aminoglycoside reduced susceptibility through RND efflux.
mphB	97.89	99.58	Chromosome	1 639 676–1 640 150 (+)	Likely intact	Predicted macrolide resistance through MPH(2′)-II-mediated phosphorylation.
emrR	100.00	100.00	Chromosome	1 836 675–1 837 205 (+)	Intact	Repressor of EmrAB–TolC; presence alone does not predict resistance.
emrA	99.83	100.00	Chromosome	1 837 332–1 838 504 (+)	Intact	Potential fluoroquinolone and multidrug reduced susceptibility through EmrAB–TolC efflux.
emrB	100.00	100.00	Chromosome	1 838 521–1 840 059 (+)	Intact	Potential fluoroquinolone and multidrug reduced susceptibility through EmrAB–TolC efflux.
tolC	100.00	100.00	Chromosome	2 239 667–2 241 154 (+)	Intact	Outer-membrane channel used by multiple multidrug efflux systems.
bacA	100.00	100.00	Chromosome	2 266 020–2 266 841 (−)	Intact	Potential bacitracin resistance through undecaprenyl-pyrophosphate recycling.
acrS	99.09	100.00	Chromosome	2 468 119–2 468 781 (−)	Intact	Regulator of AcrEF/AcrAB-associated efflux.
acrE	98.96	100.00	Chromosome	2 469 180–2 470 337 (+)	Intact	Potential β-lactam and fluoroquinolone reduced susceptibility through AcrEF–TolC efflux.
acrF	99.07	100.00	Chromosome	2 470 349–2 473 453 (+)	Intact	Potential β-lactam and fluoroquinolone reduced susceptibility through AcrEF–TolC efflux.
crp	99.21	100.00	Chromosome	2 541 437–2 542 069 (+)	Intact	Global regulator of *mdtEF* expression.
mdtE	99.57	100.00	Chromosome	2 707 148–2 708 305 (+)	Intact	Potential fluoroquinolone, macrolide and penam reduced susceptibility through MdtEF–TolC.
mdtF	98.49	100.00	Chromosome	2 708 330–2 711 443 (+)	Intact	Potential fluoroquinolone, macrolide and penam reduced susceptibility through MdtEF–TolC.
gadW	98.63	100.00	Chromosome	2 711 806–2 712 534 (−)	Intact	Regulator of *mdtEF* expression.
emrD	99.66	100.00	Chromosome	2 914 592–2 915 782 (+)	Intact	Potential multidrug reduced susceptibility through MFS efflux.
cpxA	99.78	100.00	Chromosome	3 197 674–3 199 047 (−)	Intact	Envelope-stress sensor regulating efflux-associated genes.
cpxR	99.43	100.00	Chromosome	3 199 044–3 199 742 (−)	Intact	Response regulator controlling efflux and envelope-stress genes.
soxS	99.38	99.69	Chromosome	3 394 247–3 394 569 (−)	Likely intact	Global oxidative-stress and efflux regulator.
mdtP	98.36	100.00	Chromosome	3 417 515–3 418 981 (−)	Intact	Potential efflux of acridine dyes, nucleosides and related substrates.
mdtO	99.03	100.00	Chromosome	3 418 978–3 421 029 (−)	Intact	Potential efflux of acridine dyes, nucleosides and related substrates.
mdtN	100.00	100.00	Chromosome	3 421 029–3 422 060 (−)	Intact	Potential efflux of acridine dyes, nucleosides and related substrates.

eptA	99.39	100.00	Chromosome	3 444 858–3 446 501 (−)	Intact	Potential polymyxin reduced susceptibility through phosphoethanolamine modification of lipid A.
blaEC/ampC	98.33	100.00	Chromosome	3 546 919–3 548 052 (−)	Intact	Potential penam and cephalosporin resistance through chromosomal AmpC β-lactamase activity.
**CTX-like**	95.95	100.00	Chromosome	3 685 437–3 686 325 (−)	Intact	Putative β-lactam hydrolysis by a CTX-like class A β-lactamase.
mdtM	96.35	100.00	Chromosome	3 704 277–3 705 509 (−)	Intact	Potential reduced susceptibility to fluoroquinolones, phenicols and lincosamides through MFS efflux.
robA	98.97	100.00	Chromosome	3 752 857–3 753 726 (−)	Intact	Global regulator associated with multidrug efflux and stress responses.
**CTX-like**	99.05	100.00	Chromosome	4 029 036–4 029 978 (−)	Intact	Putative β-lactam hydrolysis by a CTX-like class A β-lactamase.
ampH	98.88	100.00	Chromosome	4 153 769–4 154 926 (−)	Intact	AmpC-like β-lactamase/PBP-associated determinant.
acrB	99.59	100.00	Chromosome	4 239 802–4 242 951 (−)	Intact	Potential broad multidrug reduced susceptibility through AcrAB–TolC efflux.
acrA	99.75	100.00	Chromosome	4 242 974–4 244 167 (−)	Intact	Potential broad multidrug reduced susceptibility through AcrAB–TolC efflux.
***pbpA* (PBP2b)**	**99.37**	**100.00**	**Chromosome**	**4 400 403–4 402 304 (−)**	**Intact**	**Potential β-lactam target modification associated with PBP2b.**
kdpE	96.75	100.00	Chromosome	4 456 938–4 457 615 (−)	Intact	Transcriptional regulator associated with antimicrobial-stress responses.
mdfA	100.00	100.00	Chromosome	4 616 971–4 618 203 (+)	Intact	Potential reduced susceptibility to tetracyclines, phenicols and some biocides through MFS efflux.
msbA	98.97	100.00	Chromosome	4 700 165–4 701 913 (+)	Intact	ABC transporter associated with lipid A transport and potential multidrug export.
**CTX-like**	95.95	100.00	Plasmid	92–980 (−)	Intact	Putative plasmid-associated CTX-like class A β-lactamase.
**CTX-like**	97.41	100.00	Plasmid	79 675–80 563 (−)	Intact	Putative plasmid-associated CTX-like class A β-lactamase.
**CTX-like**	87.78	99.79	Plasmid	132 709–133 649 (−)	Likely intact	Low-identity CTX-like β-lactamase hit; allele assignment and phenotype remain uncertain.

Table S3. *Escherichia coli* strains and reference genomes used in this study. The table lists the strain designations, corresponding NCBI accession numbers, and their status as either publicly available reference genomes or novel isolates sequenced in the present study.

No.	*E. coli* strain/designation	NCBI accession used in the analysis	Status
**1**	JJ1886	CP006784.1	Reference genome
**2**	NA114	CP002797.2	Reference genome
**3**	ST131 strain EC958	HG941718.1	Reference genome
**4**	SE15	AP009378.1	Reference genome
**5**	536	GCA_000013305.1	Reference genome
**6**	CFT073	GCA_000007445.1	Reference genome
**7**	APEC O1	CP000468.1	Reference genome
**8**	S88	GCA_000026285.2	Reference genome
**9**	IHE3034	GCA_000025745.1	Reference genome
**10**	IHE3034	NC_017628.1	Reference genome
**11**	UTI89	GCF_000013265.1	Reference genome
**12**	RS218	CP007149.1	Reference genome
**13**	O7:K1	CP003034.1	Reference genome
**14**	IAI39	CU928164.2	Reference genome
**15**	PCN033	CP006632.1	Reference genome
**16**	042	NC_017626.1	Reference genome
**17**	APEC O78	CP004009.1	Reference genome
**18**	IAI1	CU928160.2	Reference genome
**19**	**ST536 O21:H9**	**JBYPZB010000001.1**	**Isolate from the present study**
**20**	K-12	GCA_000005845.2	Reference genome
